# Expression of Cystic Fibrosis Transmembrane Conductance Regulator in Ganglia of Human Gastrointestinal Tract

**DOI:** 10.1038/srep30926

**Published:** 2016-08-05

**Authors:** Ruiqi Xue, Huan Gu, Yamei Qiu, Yong Guo, Christine Korteweg, Jin Huang, Jiang Gu

**Affiliations:** 1 Department of Pathology, School of Basic Medical Sciences, Peking University Health Science Center, Beijing, China; 2 Department of Pathology, Key Laboratory of Infectious Diseases and Molecular Pathology, Guangdong Province, Collaborative and Creative Center of Molecular Pathology and Personalized Medicine, Shantou University Medical College, Shantou, China; 3 Department of Physics, University of Maryland, College Park, MD, USA

## Abstract

CF is caused by mutations of the gene encoding the cystic fibrosis transmembrane conductance regulator (CFTR) which is an anion selective transmembrane ion channel that mainly regulates chloride transport, expressed in the epithelia of various organs. Recently, we have demonstrated CFTR expression in the brain, the spinal cord and the sympathetic ganglia. This study aims to investigate the expression and distribution of CFTR in the ganglia of the human gastrointestinal tract. Fresh tissue and formalin-fixed paraffin-embedded normal gastrointestinal tract samples were collected from eleven surgical patients and five autopsy cases. Immunohistochemistry, *in situ* hybridization, laser-assisted microdissection and nested reverse transcriptase polymerase chain reaction were performed. Expression of CFTR protein and mRNA was detected in neurons of the ganglia of all segments of the human gastrointestinal tract examined, including the stomach, duodenum, jejunum, ileum, cecum, appendix, colon and rectum. The extensive expression of CFTR in the enteric ganglia suggests that CFTR may play a role in the physiology of the innervation of the gastro-intestinal tract. The presence of dysfunctional CFTRs in enteric ganglia could, to a certain extent, explain the gastrointestinal symptoms frequently experienced by CF patients.

Cystic fibrosis (CF) is a multiorgan autosomal recessive disorder caused by mutation of the cystic fibrosis transmembrane regulator (*CFTR*) gene^1^,^2^,^3^. The *CFTR* gene encodes a cAMP-regulated chloride channel protein present in the apical membrane of epithelial cells. Over 1900 mutations have been identified in the *CFTR* gene, but 70% of CF patients have has a homozygote deletion of three base pairs in the *CFTR* nucleotide sequence resulting in the absence of the phenylalanine residue at position 508 of the CFTR protein^4^,^5^.

The primary sites where CF manifests itself include the respiratory tract, digestive tract, urogenital tract, and sweat glands^6^,^7^. In these organs the malfunction of the mutated ion channel leads to fluid and electrolyte transportation disorders along with obstructed ducts, atrophic epithelia, gland hypertrophy and ultimately results in inflammation and fibrosis^8^,^9^,^10^,^11^,^12^,^13^. CFTR was originally thought to be exclusively expressed by epithelial cells. However, recent studies have shown CFTR expression in neurons of the human central nervous system, including the brain and spinal cord^14^,^15^,^16^,^17^,^18^. In addition, widespread presence of CFTR has been demonstrated in the peripheral nervous system, including human spinal, sympathetic and paracervical ganglia^14^,^15^,^16^,^19^,^20^. These findings suggest that CFTR plays a significant role in the physiology of the central and peripheral nervous system.

The enteric nervous system (ENS) is regarded as the intrinsic nervous system of the tractus gastrointestinalis^21^,^22^. It is composed of two plexuses embedded in the wall of the gastrointestinal tract, i.e. the myenteric plexus and the submucosal plexus^21^,^22^. The ENS is connected to the central nervous system through sympathetic and parasympathetic nerves. It regulates motility, glandular secretions, fluid transport, and local blood flow, among others^21^. In addition to the tractus gastrointestinalis, the ENS also exists in the walls of the pancreas and biliary tract, but these organ systems were not investigated in the present study.

CF patients may suffer from various gastrointestinal tract diseases, including the distal intestinal obstruction syndrome, meconium ileus of the newborn, intussusceptions, small bowel bacterial overgrowth, rectal prolapse, gastroparesis and gastroesophageal reflux^11^,^23^,^24^. The majority of these diseases are is causally related to the malfunction of the CFTR protein in the epithelial cells of the gastrointestinal tract and pancreas resulting in enzyme insufficiency and thickening of gastrointestinal secretions^25^,^26^,^27^,^28^. However, given its crucial role in the control of motility, and exocrine and endocrine secretions in the tractus gastrointestionalis, it is conceivable that abnormalities in the ENS might also contribute to the pathogenesis of the gastrointestinal diseases found in CF. In this study, we aimed to investigate the distribution and expression of CFTR protein and mRNA in ganglion cells of the human ENS. Widespread expression of CFTR was demonstrated in the ENS, which could provide an additional explanation for the gastrointestinal symptoms in CF.

## Materials and Methods

### Human Tissue Collection

Human tissue samples of gastrointestinal tracts were obtained from a total of 16 patients, 11 from patients undergoing surgery and 5 autopsy cases from the Peking University Third Hospital (Beijing, PR China) and Shantou Medical University Hospital (Shantou, PR China). None of the subjects was diagnosed with any disease of the nervous system or CF. All methods were carried out in accordance with the approved guidelines of the Institutional Animal Care and Use Committee Guide of Peking University. All experimental protocols were approved by Peking University. The study received local hospital ethics committee approval. Informed consent was obtained from all patients. General information about these cases is listed in Table 1.

### Tissue Preparation

The autopsied bodies were kept at 4 °C, and the autopsies were performed within 48 hours after death. For immunohistochemistry and *in situ* hybridization, samples were fixed in 4% formalin solution after removal for 24 hours and then embedded in paraffin blocks. For reverse transcription-polymerase chain reaction (RT-PCR), fresh tissue samples were immediately snap-frozen in liquid nitrogen and stored at −80 °C until use. The segments evaluated in this study include the stomach, duodenum, jejunum, ileum, cecum, appendix, colon and rectum. In each surgical case, the appropriate consent for the use of tissues was obtained. Samples were taken from macroscopically unaffected areas that were determined by visual inspection.

### Immunohistochemistry localization

CFTR expression was localized, using immunohistochemistry on consecutive formalin-fixed paraffin-embedded tissue sections (4 *μ*m) which preserve good morphology for neuron identification and CFTR protein expression as following: (1) The tissue sections were deparaffinized in xylene and rehydrated in gradient ethanol, incubated in 70% ammonia-ethanol at room temperature for 30 min to remove formalin deposition and then washed in 0.01 M phosphate-buffered saline (PBS). (2) Antigen retrieval was performed by heating the slides at 96 °C in a sodium citrate buffer (pH 6.0) for 15 min^29^. (3) After cooling to room temperature, slides were rinsed in 0.01 M PBS, then again in 3% hydrogen peroxide incubation for 30 min to quench the endogenous peroxidase activity. They were then washed with 0.01 M PBS 3 times for 3 min each. (4) Primary mouse anti-human monoclonal antibodies to CFTR (1:100; Zymed Laboratories, South San Francisco, CA, USA) and neurofilament (NF, 1:100; Dako, Copenhagen, Denmark) were added to 2 consecutive sections to identify CFTR-positive cells and ganglion cells, respectively, and incubated overnight at 4 °C. PBS was used in place of the primary antibody for the negative control sections. (5) Horseradish-peroxidase-conjugated anti-mouse/rabbit IgG (PV9000 immunohistochemistry kit; Zymed Laboratories) and horseradish-peroxidase conjugated anti-goat IgG (1:1000; Jackson, West Grove, PA, USA) were added and incubated at room temperature for 20 min as the secondary antibody. (6) After being washed in PBS, 3,3′-diaminobenzidine (DAB; Zymed Laboratories) was used to visualize antigen localization. All slides were counterstained with hematoxylin. The results of immune staining on neurofilament (NF) on adjacent sections were compared to examine whether the CFTR-containing cells were neurons.

### Laser-assisted microdissection, RNA extraction and nested RT-PCR

Because of the strong positive expression of CFTR in gastrointestinal tract epithelial cells, we performed a laser-assisted microdissection (LMD) to ensure the isolation of only gastrointestinal ganglion cells for RNA extraction followed by nested RT-PCR, to avoid possible contamination of nearby epithelial cells. The frozen tissue samples of human gastrointestinal tract segments were sectioned (10 *μ*m) and mounted on pretreated slides (LCM DNase-free, RNase-free, PEN-membrane slides, Leica Microsystems, Wetzlar, Germany). The slides were then quickly fixed in 70% ethanol for 1 min, rinsed in DEPC-treated water for 30 s, stained with DEPC-treated hematoxylin for 1 min, and rinsed in DEPC-treated water (pH 8.0) twice for 30 s each time. All of the chemicals were prepared with DEPC-treated water. The ganglion cell groups were identified by their morphology, and were isolated from the frozen tissue sections using the Leica Microdissection System (Leica LMD 6000 B, Leica Microsystems). The microdissected target ganglia were then collected in an Eppendorf tube. Smooth muscle cell groups were captured and used to exclude the expression of CFTR in smooth muscle cells. Epithelial cell groups were also captured and used as positive controls. The procedure was performed in accordance with the manufacturer’s instructions.

RNA extraction was performed using the RNeasy Micro Kit (Qiagen, Cologne, Germany) to extract total RNA from the dissected ganglion cells. The reverse transcription was carried out with the Super Script III cDNA Kit (Invitrogen, CA, USA) following the manufacturer’s instructions. Briefly, 5 *μ*g of total RNA was extracted from the dissected tissue and reverse transcription reactions were performed using random primers at 55 °C for 60 min.

The sequences of the CFTR primers were obtained from the GenBank (acc. No. NM-031506). Nested RT-PCR was used to ensure the specificity of the results as two pairs of primers were employed. The primers for nested PCR were as follows: external 5′-CACTGCTGGTATGCTCTCCA-3′ (sense), and 5′-AATGAATGGCATCGAAGAGG-3′ (antisense); internal 5′-CACTGCTGGTATGCTCTCCA-3′ (sense), and 5′-ACCGAAAGACAACAGCATCC-3′ (antisense). The PCR products span one intron. 18S was amplified as an endogenous reference with the following primers: 5′-AAACGGCTACCACATCCAAG-3′ (sense), and 5′-CCTCCAATGGATCCTCGTTA-3′ (anti-sense). The final amplifying products of CFTR and 18S were 179 bp and 155 bp. Nested PCR was performed with Taq Polymerase (NEB, Nebraska, US). The first amplification with the external primers proceeded for 40 cycles of 30 s at 94 °C, 40 cycles of 30 s at 55 °C, and 40 cycles of 1 min at 72 °C. The final cycle was followed by an extension period of 10 min at 72 °C. The second amplification round with the internal primers proceeded in a manner identical to the first amplification round.

### Plasmid construction, sequence analysis and cRNA probe preparation

To perform sequence analysis and generate the cRNA probe, we used a specifically designed CFTR RT-PCR product, as described before^17^,^18^,^30^. Human brain tissue RNA extraction was carried out using oligo (dT) primers by a cDNA synthesis kit (#K1621, Fermentas; Lithuania). The PCR was then performed with pairs of specific primers for nested RT-PCR. They were encoded as follows: 5′-CCCTTCGGCGATGTT-3′, 5′-CAGGAAACCAAGTCCACAG-3′ (external); and 5′- AGGAGGAACGCTCTATCG-3′, 5′-GCAGACGCCTGTAACAAC-3′ (internal). The final amplification product of CFTR was 328 bp and encompassed two splicing events. The first amplification of PCR was performed for 40 cycles of 1 min at 94 °C, 40 cycles of 30 s at 72 °C, and 40 cycles of 30 s at 52 °C; the second amplification round was for 40 cycles of 1 min at 94 °C, 40 cycles of 30 s at 72 °C, and 40 cycles of 30 s at 54 °C. In both reactions, the first PCR cycle was preceded by an incubation period of 10 min at 94 °C, and the final cycle was followed by an extension period of 5 min at 72 °C. Five *μ*g of the product was separated on 2% agarose gel by electrophoresis, extracted from the gel using the Gel DNA Extraction kit (Tiangen Biotech, Beijing, China), and then subcloned into the pGM-T vector by T4 ligase (Tiangen Biotech). The plasmid was then transfected into Escherichia coli XLl-Blue. X-gal/ isopropyl-b-D-thiogalactopyranoside and ampicillin (100 *μ*g/mL) double selections were performed.

Sequencing was carried out to determine the identity of the CFTR gene. Plasmid, extracted from the positive clone, was linearized with either the SalI or NcoI restriction enzyme, and an *in vitro* transcription reaction was performed to generate the cRNA probe. Anti-sense cRNA probes were generated with Sp6 RNA polymerase, and sense probes were generated with T7 RNA polymerase. The probes were labeled with a Digoxigenin RNA Labeling Mix (Roche Molecular Biochemicals; Mannheim, Germany) for *in situ* hybridization.

### *In situ* hybridization localization

To determine the *CFTR* mRNA localization in human gastrointestinal ganglia, we performed *in situ* hybridization on consecutive formalin-fixed paraffin-embedded tissue sections (4 *μ*m) with a specific cRNA anti-sense probe against the CFTR gene prepared as described above. All the solutions used were prepared with diethyl-pyrocarbonate (DEPC)-treated water to avoid mRNA degradation. The tissue sections were deparaffinized in xylene, rehydrated in gradient ethanol, incubated in 1 N HCl at room temperature for 10 min to increase permeability and washed in 0.01 M phosphate-buffered saline (PBS). The slides were heated to 96 °C in sodium citrate buffer (pH 6.0) for 15 min^29^. After being cooled to room temperature, and rinsed in 0.01 M PBS, the slides were fixed in 4% paraformaldehyde for 10 min, washed and dehydrated in 90% ethanol for 15 s, and hybridized overnight at 45 °C with the specific digoxigenin-labeled CFTR cRNA probe. The slides were then washed three times, first with 2× standard saline citrate (SSC) plus 50% formamid, then with 2× SSC, and finally with 0.1× SSC at 37 °C for 15 min each. After blocking with normal horse serum (1:100) at room temperature for 1 hour, the sections were incubated with an alkaline phosphatase-labeled anti-digoxigenin antibody (1:500; Roche Diagnostics, Pensberg, Germany) for 1 hour. Nitro blue tetrazolium/5-bromo-4-choloro-3-indolyl phosphate (NBT/BCIP; Promega Corp., Madison, Wis.) was used to visualize the reaction staining, which resulted in a purple-blue signal. All slides were counterstained with methyl green. The slides were incubated with a corresponding sense probe or with hybridizing solutions only as negative controls.

To identify the cell type that expressed *CFTR* mRNA, immunostaining with antibody to NF was performed on tissue sections consecutive to the ones on which CFTR *in situ* hybridization staining was performed. The NF immunostaining results were compared with the *CFTR in situ* hybridization results on consecutive sections to investigate whether *CFTR* mRNA positive cells were NF-positive neurons.

## Results

### CFTR expression in human enteric ganglia by IHC

The human enteric ganglia were identified based on their location and morphology. They were composed of small cell groups of enteric neurons, glial cells and nerve fibers. The number, shape, size and orientation of the neurons in each ganglion varied in the different gastrointestinal segments.

**Figure 1:**
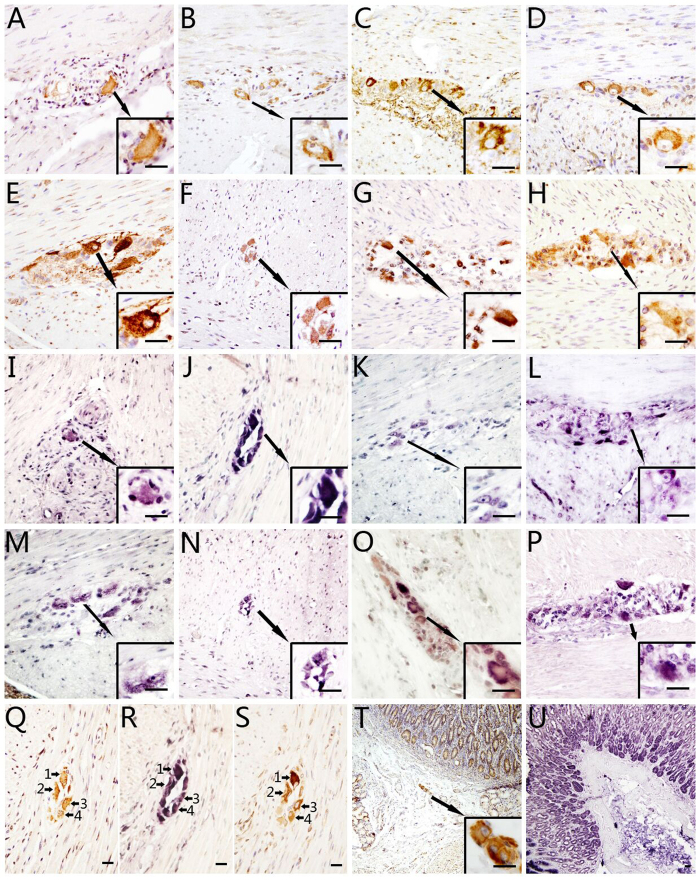
Cystic fibrosis transmembrane conductance regulator (CFTR) expression in the neurons of human enteric ganglia as detected by immunohistochemistry (IHC) and *in situ* hybridization (ISH). (**A**–**H**): IHC results for CFTR in human gastrointestinal ganglia. (**A**–**H**) show segments of stomach (**A**), duodenum (**B**), jejunum (**C**), ileum (**D**), cecum (**E**), appendix (**F**), colon (**G**) and rectum (**H**). Lower right square insert in each figure shows the higher power image of indicated area of the lower power view. Each photograph shows cell groups of enteric ganglia between two layers of smooth muscle of different orientations. The brown positive signals developed with DAB show positive CFTR staining. CFTR-positive signals are localized to the cytoplasm of the neurons and to some dendrites, axons and nerve fibers. The nuclei and nucleoli lack positive signals. Smooth muscle cells and glial cells are also negative. (**I**–**P**): ISH results for CFTR in human enteric ganglia. (**I**–**P**) show segments of stomach (**I**), duodenum (**J**), jejunum (**K**), ileum (**L**), cecum (**M**), appendix (**N**), colon (**O**) and rectum (**P**). Lower right square insert in each figure shows the higher power image of indicated area of the lower power view. The purple-blue positive signals developed with NBT/BCIP show *CFTR* mRNA ISH staining. CFTR-positive signals are localized to the cytoplasm of the neurons, some of the nuclei and nucleoli, dendrites, axons and nerve fibers. Smooth muscle cells are negative. (**Q–S**): Higher power image for CFTR IHC and ISH results and consecutive sections of IHC with antibodies to neurofilament. (**Q**–**S**) consecutive sections show the CFTR IHC (**Q**), ISH (**R**) and the NF IHC (**S**) results of a same ganglion, and numbered arrows indicate the same cells. Positive NP signals are observed in CFTR IHC and *CFTR* mRNA ISH positive cells on consecutive sections, indicating that the CFTR positive cells are neurons. (**T**): Positive control of CFTR IHC: gastric epithelium. Lower right square insert shows the higher power image of a submucosal plexus. The brown positive signals developed with DAB show positive CFTR staining, same as the epithelium. (**U**): Positive control of *CFTR* mRNA ISH in gastric epithelium. Bar = 20 *μ*m.

CFTR immunoreactivity was detected in the cytoplasm and dendrites of all neurons of the human enteric ganglia by IHC, both in myenteric and submucosal plexus. Consecutive sections showed the presence of positive NF (neurofilament, specific neuronal cell marker) IHC signal in all CFTR-positive cells. The smooth muscle cells and glial cells showed negative immunostaining for CFTR and NF. The specificity of IHC was established in parallel control experiments. In the positive control, strong CFTR-positive signals were detected in human gastric epithelium. In the negative controls, no specific signal was observed. In all segments of the tractus gastrointestinalis, the expression and distribution of CFTR were similar (Fig. 1). No obvious differences were observed either among the different segments of the gastrointestinal tract of each case, or among the different cases.

### *CFTR* mRNA expression in human gastrointestinal ganglia by ISH

*CFTR* mRNA expression was detected and localized by *in situ* hybridization. Positive *CFTR* mRNA anti-sense signals were found in the cytoplasm and dendrites of all neurons of the enteric ganglia. Positive signals were detected in all of the gastrointestinal segments examined. The distribution pattern was identical to that of CFTR protein as detected by IHC. In some cases nuclei also showed positive hybridization signals. No signal was observed in either smooth muscle cells or glial cells. IHC with antibody to NF on consecutive sections confirmed that the *CFTR* mRNA positive cells were indeed neurons. In gastric epithelium, which was used as positive control, strong *CFTR* mRNA signals were detected. In the negative controls, no specific signal was observed when the specific probe was replaced by the *CFTR* sense probe or hybridization solution. The expression and distribution of *CFTR* mRNA displayed the same pattern among the various segments of the gastrointestinal tracts studied (Fig. 1).

### Localization and detection of *CFTR* mRNA in ganglion cells by LMD-assisted nested RT-PCR

**Figure 2:**
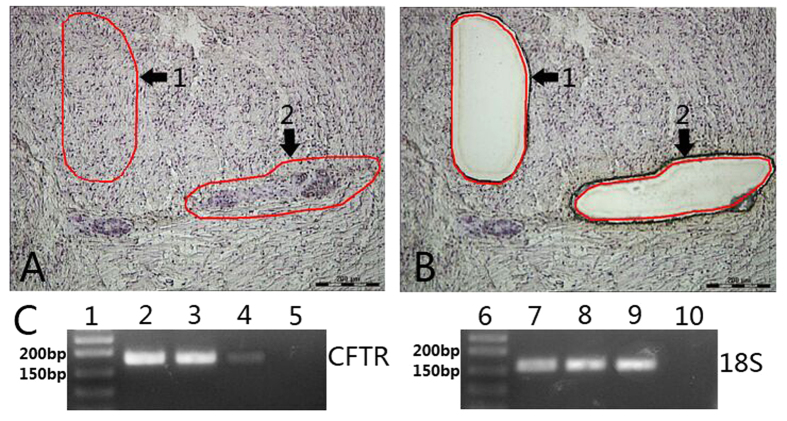
Laser-assisted microdissection (LMD) and nested RT-PCR of CFTR in human enteric ganglia. (**A**,**B**) show the location where the LMD dissected the collections of ganglion cells (**A2**) and smooth muscle cells (**A1**). (**A**) is photograph taken before, and (**B**) after LMD. Numbered arrows indicate the same collection of cells. (**C**) shows the expression of *CFTR* mRNA by nested RT-PCR. Lane 1, DNA marker; Lane 2, enteric ganglia; Lane 3, colon mucosa (positive control); Lane 4, smooth muscle; Lane 5, water (negative control); Lane 6, DNA marker; Lane 7, enteric ganglia; Lane 8, colon mucosa (positive control); Lane 9, smooth muscle; Lane 10, water (negative control). The CFTR product is 179 bp, and the 18S product is 155bp. Bar = 200 *μ*m.

As CFTR is known to be extensively expressed in the epithelium throughout the gastrointestinal tract, we performed LMD to carefully isolate enteric ganglia without epithelial cells (Fig. 2). Following isolation of the ganglion cells, nested RT-PCR was performed. The CFTR product of 179 bp was identified by agarose gel electrophoresis (Fig. 2). In addition, NF mRNA was successfully amplified confirming that ganglionic neurons had indeed been captured by LMD, whereas lack of CK mRNA amplification indicated that solely ganglion cells had been isolated. Smooth muscle cells were captured to exclude the expression of CFTR and showed a much weaker positive PCR product. Colon epithelial tissue was used as positive control and showed a PCR product of the expected size. In the negative control no amplification signal was detected. Sequence analysis was in 100% agreement with the human *CFTR* mRNA sequence as encoded by the NCBI.

## Discussion

In this study we demonstrated CFTR expression in human enteric ganglia, which could offer a better understanding of the functions of CFTR in the normal physiology of the ENS, as well as in CF patients.

Originally the epithelial cell was regarded as the only cell type expressing CFTR. The CFTR gene with its gene product was identified in 1989 and its expression was detected in the epithelial cells of those organs most severely affected in CF, including the lungs, intestines, kidney, pancreas and liver^25^,^31^,^32^,^33^. In the tractus gastrointestinalis CFTR was found localized to the mucosal epithelial cells of the stomach, duodenum, jejunum, ileum and colon, with crypt cells expressing more CFTR than villus cells^34^,^35^. However, in the years following the discovery of CFTR several other cell types were found to express CFTR, such as fibroblasts, neutrophils, lymphocytes, macrophages, and mast cells^36^,^37^. In respect to the human nervous system, CFTR expression was detected in the hypothalamus^15^. Yet recent studies have demonstrated a more widespread expression of CFTR in both the central and peripheral nervous system, including various regions of the brain, as well as spinal, cerebellum, sympathetic ganglia, paracervical ganglia and trigeminal ganglion^17^,^18^,^38^,^39^,^40^. Reznikov *et al*. reported CFTR expression and activity in Schwann cells by studying new born CFTR-deficient pigs, suggesting that nervous system abnormalities in patients with CF might be directly related to the loss of *CFTR*^40^. In the present study we investigated CFTR expression in the ganglia of the ENS in various segments of the human gastrointestinal tract, including the stomach, duodenum, jejunum, ileum, cecum, appendix, colon and rectum. The results of IHC, ISH and RT-PCR, all convincingly demonstrate the presence of *CFTR* mRNA and protein in both the myenteric and submucosal plexuses. Immunohistochemistry with antibodies to CFTR and NF and *in situ* hybridization with a CFTR mRNA anti-sense probe on consecutive sections demonstrated that the cells expressing CFTR were NF-positive neurons. As the enteric ganglia are in close proximity to the mucosal epithelial cells, which abundantly express CFTR, LMD was performed prior to RT-PCR to isolate only neurons. Successful amplification of NF mRNA and lack of amplification of CK mRNA confirmed that neurons were captured, but not epithelial cells.

The extensive expression of CFTR in both the central nervous system and the peripheral nervous system including the ENS suggests that this protein may play a role in the normal functioning of the nervous system. CFTR might be involved in neuronal physiology through several mechanisms, as discussed previously by Guo *et al*.^17^,^18^, with the most important ones being the following: maintenance of the steady-state of intracellular electrolytes^41^, regulation of membrane recycling^42^,^43^,^44^,^45^,^46^,^47^, modulation of membrane traffic^48^,^49^, governing the efflux of gluthatione^50^,^51^, regulation of neuropeptide secretion^15^, and functioning as a neuromodulator and cell signaling molecule. Consequently, mutation of CFTR could theoretically lead to neuronal dysfunctioning.

The ENS constitutes together with the parasympathetic and sympathetic nervous systems the autonomous nervous system. Autonomous neuropathy appears to exist in patients with CF^52^, as illustrated by decreased cardiovascular sensitivity to β-adrenergic stimulation^12^,^53^ and abnormal α-adrenergic and cholinergic regulation of the pupils^53^,^54^. Whether autonomic dysfunction of the gastrointestinal tract frequently occurs in CF patients is not clear, as symptoms might be non-specific and accurate tests to determine abnormalities are not readily available^52^. The main etiological factors of autonomic neuropathy in CF are considered to be metabolic and nutritional^52^. Given the expression of CFTR in the ENS together with the previously established CFTR expression in the sympathetic ganglia and paracervical ganglia, it is conceivable that malfunction of CFTR in the ganglionic neurons also contributes to the occurrence of autonomic neuropathy in CF.

Gastrointestinal manifestations are frequently observed in CF patients and include delayed gastric emptying, gastritis, distal intestinal obstruction syndrome, meconium ileus of the newborn, intussusceptions, fibrosing colonopathy, and rectal prolapse, among others^8^,^10^,^11^,^23^,^24^,^28^,^55^,^56^,^57^. Some of these manifestations have a direct causal relation to the malfunction of the CFTR in epithelial cells of gastrointestinal tract and pancreas, such as the distal intestinal obstruction syndrome and meconium ileus, whereas others, such as fibrosing colonopathy and gastritis, are regarded as secondary complications of the disease or its therapy^24^. As CFTR is responsible for anion transport^58^,^59^, its dysfunction causes abnormal water and electrolyte secretion resulting not only in thick and adherent fluids in the intestines but also in the development of mucous and acidic enzyme secretions in the pancreas causing destruction of pancreatic tissue with ensuing exocrine pancreatic insufficiency leading on its turn to malabsorption in the gastrointestinal tract^57^,^60^. In light of the regulatory role of the ENS in motility, water and electrolyte flow and endocrine secretions in the gastrointestinal tract^21^, it appears, however, not unlikely that ENS abnormalities caused by dysfunctional CFTR may also play a role in the pathogenesis of gastrointestinal diseases in CF.

In this context, it is of interest to note that immaturity of the myenteric plexus appeared to be the main etiological factor in newborns with meconium ileus without CF^61^,^62^. In addition, myenteric ganglionitis, aganglionosis, and neuronal dysplasia have all been found in CF patients with distal intestinal obstruction syndrome and meconium ileus^63^,^64^,^65^. These findings support the assumption that abnormalities of enteric ganglia might induce dysfunction of the gastrointestinal tract.

In conclusion, our study provides evidence of CFTR expression in the neurons of the ENS. Widespread distribution of CFTR in enteric ganglia throughout the gastrointestinal tract suggests that this protein might exert a role in maintaining the normal structure and physiological functions of enteric ganglion cells. In addition, the presence of dysfunctional CFTR in the ENS may have adverse effects on the gastrointestinal tract. Further research is required to clarify the exact function of CFTR in the ENS and the implications of dysfunctional CFTR in the enteric ganglia for patients with CF.

## Additional Information

**How to cite this article**: Xue, R. *et al*. Expression of Cystic Fibrosis Transmembrane Conductance Regulator in Ganglia of Human Gastrointestinal Tract. *Sci. Rep.* **6**, 30926; doi: 10.1038/srep30926 (2016).

## Figures and Tables

**Table 1:** Characteristics of the 16 subjects studied.

Case No.	Sex/Age (y)	Pathological diagnosis	Segments investigated in the study							
			Stomach	Duodenum	Jejunum	Ileum	Cecum	Appendix	Colon	Rectum
1	M/41	Ileum interstitialoma				√				
2	M/53	Pancreatic head adenocarcinoma	√							
3	F/61	Pancreatic head ductal adenocarcinoma		√						
4	F/36	Colonic adenocarcinoma					√	√		
5	F/60	Colonic adenocarcinoma						√	√	
6	M/77	Rectal adenocarcinoma								√
7	M/66	Lesser curvature adenocarcinoma	√	√						
8	M/50	Duodenal adenocarcinoma		√	√					
9	M/35	Colonic adenocarcinoma				√			√	
10	F/52	Rectal adenocarcinoma							√	√
11	M/71	Rectal adenocarcinoma							√	√
12	F/28	Amniotic fluid embolism	√			√		√	√	
13	M/25	Epidemic cerebrospinal meningitis	√		√				√	
14	F/74	Hypertensive heart disease	√			√		√	√	
15	M/81	Dissecting aneurysm of the abdominal aorta	√	√		√			√	√
16	F/78	Acute pulmonary embolism	√	√	√		√	√	√	√
